# Do farmers perceive risks of fraudulent pesticides? Evidence from Saudi Arabia

**DOI:** 10.1371/journal.pone.0239298

**Published:** 2020-09-28

**Authors:** Hazem S. Kassem, Bader Alhafi Alotaibi

**Affiliations:** 1 Department of Agricultural Extension and Rural Society, King Saud University, Riyadh, Saudi Arabia; 2 Department of Agricultural Extension and Rural Society, Mansoura University, Mansoura, Egypt; University of Lincoln, UNITED KINGDOM

## Abstract

The increasing number of fraudulent pesticides on the market not only constitutes a major threat to sustainable agriculture but can also have adverse consequences for the environment and human health. The purpose of this study is to assess farmers’ risk perception with regard to fraudulent pesticides and to establish the determinants of their perception. Data were collected through structured questionnaires from 370 farmers from the eastern region in Saudi Arabia. The findings showed that farmers had a high perception of physical, legal, social, and physiological risks of counterfeit pesticides, while they had a moderate perception of agri-environmental risks (M = 3.47, SD = 0.72) and economic risks (M = 3.52, SD = 1.11). Moreover, 73.5% of farmers reported that they had purchased fraudulent pesticides in the last three years. The results of the t-test revealed that the number of farmers who had purchased fraudulent pesticides was significantly higher than the number of farmers who had not purchased such pesticides regarding the perception of the majority of risks, except for physical risk. Multivariate regression analyses showed that age, farm size, farming experience, extension contact, and purchased fraudulent pesticides were significantly associated with risk perception. The findings suggest that awareness campaigns on recognizing fraudulent pesticides among farmers are needed, as well as policy measures, to combat counterfeiting in the agricultural sector in cooperation with other stakeholders.

## Introduction

Pesticides are among the most regulated products in the world. However, the trade of fraudulent pesticides has increased in scope and magnitude in recent years [1]. According to a European Union Intellectual Property Office (EUIPO) report [2], the production of fraudulent pesticides costs EU European agrochemical producers about €1 billion, accounting for 8.3% of the legitimate revenue each year. This equates to a loss of 1749 jobs in the pesticide sector in the EU. When the knock-on effects of fraudulent pesticides in the marketplace are taken into account, 7993 jobs are lost in the EU economy. The total yearly loss of government revenue as a result of fraudulent pesticides in this sector across the EU in terms of taxes and social contributions is estimated to be €300 million. Germany, France, and Italy have suffered the most in terms of lost sales, according to the EUIPO’s report. In the case of Saudi Arabia, a report of the Ministry of Commerce showed that the sector of pesticides and agrochemicals ranked first among other sectors regarding the percentage of fraudulent pesticides inspected in 2018 [3]. According to this report, illegal and counterfeit pesticides accounted for 17% of the market share of plant protection products in 2018 compared to 14% in 2015.

There are three main clusters of fraudulent pesticides in the pesticide market, including (i) fake products, which are deliberately and fraudulently mislabeled to look like the genuine product. These products might contain chemicals that are either banned or restricted, the wrong ingredients (quality and quantity) [4], and often sold in simple packs, such as plain bottles with minimal labeling describing their use, but no health or environmental precautions [5], (ii) illegal imports/sales of non-authorized products or registered products from non-registered sources by non-authorized distributors [6], and (iii) counterfeits, sophisticated copies of legitimate branded products usually with high quality labelling and packaging [5]. The purchasing behavior of fraudulent pesticides is classified into two main categories: deceptive, when a farmer thinks that the pesticide is original and he/she is not aware of buying unauthorized and fake products, and non-deceptive, when a farmer purchases fraudulent pesticides knowingly and intentionally [7].

There are different potential risks associated with fraudulent pesticides. At an agricultural production level, such pesticides can severely damage crops, resulting in a decreased yield or destroying a field, and pose severe health risks to the farmers through exposure during application [8]. Furthermore, residues of unknown and untested substances in foods can negatively affect consumers’ health [9,10]. The use of fraudulent pesticides also has environmental consequences. Firstly, many active substances and other constituents used in counterfeit and illegal pesticides contain highly toxic impurities, which can pose a risk to the water and soil quality and the health of biodiversity [11]. Secondly, the production of fraudulent pesticides may subvert environmental regulations, leading to the production processes and waste contaminating the land, air, and water [12]. Thirdly, the destruction of fraudulent pesticides can result in more landfill waste or toxic fumes from incineration [6]. On the other hand, fraudulent pesticides have adverse impacts on the plant protection industry, including a loss of sales, damage to one’s reputation, patent and trademark infringement, and the undermining of industry stewardship activities [13]. Moreover, increased cases of such pesticides can result in economic damage to governments. This includes job losses, stifle innovation and competitiveness, lost taxes and levies from the sale of genuine products, and a reduction in the public confidence in the government’s ability to effectively regulate the agricultural sector [14].

Farmers are both the producers and consumers of food, and as such, have a dual role in food safety: they both generate risks and are exposed to them [15]. Consequently, increasing farmers’ perception of the associated risks of using fraudulent pesticides in pest control is one of the main approaches employed to combat fraudulent pesticides at the micro level [16]. According to Jin et al. (2016) [17], risk perception exerts a strong influence on the vast scope of farmers' decisions, reducing uncertainty and avoiding unfavorable consequences of their decisions. Different studies confirm that farmers differ in their risk assessments of the same object, depending on individual and situational factors [18–20]. Farmers perceive different levels and various dimensions of risks associated with fraudulent pesticides utilization [9]. Physical, environmental, psychological, economic, social, and legal risks were among the first risk facets [21].

Addressing the frauds issue is a collective responsibility. In this regard, Yao’s work is one of the most important examples of successful private-sector-led efforts to combat fraudulent pesticides in West Africa in collaboration with a broad array of interested stakeholders [22]. The Yao’s strategy depends upon make all stakeholders realize that there is a problem, ensure stakeholders can recognize fraudulent pesticides, ensure stakeholders can take proper actions, and lobby/influence decision makers. The activities including, awareness creation & communication methods, training and building capacity programs for all stakeholders, and advocacy towards governmental authorities.

Despite the fact that several studies have been undertaken to investigate farmers’ perception and awareness of pesticide use management and safety measures [17,23–30], very few studies have assessed the perception of fraudulent pesticides and its determinants among farmers, in particular, in the context of Saudi Arabia. Accordingly, there is a research gap in terms of farmers’ perception level of different risk facets of fraudulent pesticides and factors influencing this perception. To try to fill this gap, the current study aimed to (i) identify farmers’ risk perception regarding fraudulent pesticides, (ii) determine the differences in risk perception between farmers who had purchased fraudulent pesticides and those who had not, and (iii) explore the factors influencing farmers’ risk perception.

## Methods

### Study area

The Al-Ahsa governorate, located in Eastern Saudi Arabia, is the biggest of the eight governorates in the eastern province. This governorate was selected as a site for the current study, as it accounts for around (68%) of the total agricultural land in the region [31]. Furthermore, the geographic proximity of this governorate to the research team facilitated communication and collection of the needed information. Al Ahsa has one of the largest oases in the world, with more than three million date palms in a total area of 16,000 hectares. The cropping pattern in the Al-Ahsa governorate mainly consists of, besides palm trees, tomatoes, okra, grape, pomegranate, and rice [32].

### Sampling and data collection

Four villages in the governorate were randomly selected for the purposes of this study: Al-Hota, Al-Hilala, Al-Remila, and Al-Monizala. The study population consisted of all farmers registered in the records of the Agricultural Directorate of Al Ahsa during the agricultural season of 18/2019 in these villages (n = 4712). Using the formula of Krejcie and Morgan (1970) [33], a random sampling method was employed to select 388 farmers from the four villages. A total of 370 completed the interview, resulting in a response rate of 95.3%. The survey was conducted from October to December 2019, following ethical approval from King Saud University. Farmers were first provided with the purpose of the research and asked for their consent to participate. Those who agreed to participate completed a face-to-face interview using a structured questionnaire.

### Instrument

The questionnaire comprised three sections. Section one covered the socio-economic characteristics of respondents. Section two focused on farmers’ exposure to fraudulent pesticides. Twenty risk items related to fraudulent pesticides were identified from the literature (section 3). The perception of risks of the sampled farmers was measured on a five-point Likert scale (1 = “strongly disagree,” 2 = “disagree,” 3 = “neutral,” 4 = “agree,” and 5 = “strongly agree”). The validity of the risk items was established using a panel of 10 experts from plant protection and agricultural extension departments at King Saud University. In addition, the internal reliability of the scale was examined using Cronbach’s alpha, and was found to be 0.82.

### Data analysis

Descriptive statistics, such as the frequency, percentages, mean, and standard deviation, were applied to describe the study’s variables. An independent sample t-test was used to compare farmers who had purchased fraudulent pesticides and farmers who had not, regarding their risk perception. Moreover, multivariate regression analysis was used to examine the determinants associated with the risk perception of fraudulent pesticides. Six determinants were selected in the model: age, education, farming experience, extension, farm size, and purchased fraudulent pesticides. However, regression analysis requires numerical variables, so some variables were converted into dummy variables, as follows: education (had at least basic education = 1, other = 0), extension (extension as a source of information = 1, other = 0), and purchased fraudulent pesticides (non-purchased = 1, purchased = 0).

## Results

### Socio-economic profile of the respondents

Most respondents (61.1%) were aged between 45 and 60 years, with an average age of 56.1 years (Table 1). More than one-third (38.1%) had no formal education and only a small proportion (14.6%) had a university degree. The mean farming experience of respondents was 25.9 years, and almost one-half (50.8%) had between 16 and 30 years of farming experience. Regarding the farm size, most farmers (60.8%) had a farm smaller than 1 hectare, with an average farm size of 2.84 hectares. Farmers managed more than one farming activity in the study area. Vegetables were the main farming activity, being cultivated by 60.5% of the respondents, followed by crops (55.9%) and fruits (37.6%). For information sources, the majority of farmers (79.2%) obtained their information on pesticides from other farmers, while pesticide retailers ranked second, with a percentage of 70.5%.

**Table 1:** Socio-economic characteristics of the respondents.

Variable	Frequency	%
Age (Mean = 56.1, SD = 9.4)		
<45 years	32	8.6
45–60 years	226	61.1
>60 years	112	30.3
Education		
Illiterate	141	38.1
Read and write	105	28.4
Basic education	28	7.6
Secondary school	42	11.4
University	54	14.6
Farming Experience (Mean = 25.9, SD = 10.5)		
<16 years	76	20.5
16–30 years	188	50.8
>30 years	106	28.6
Farm size (Mean = 2.84, SD = 1.98)		
<1 hectare	225	60.8
1–3 hectare	73	19.7
>3 hectares	72	19.5
Type of farming activities*		
Crops	207	55.9
Vegetables	224	60.5
Fruits	139	37.6
Information sources on pesticides*		
Other farmers	293	79.2
Extension	135	36.5
Pesticide retailer	261	70.5
T.V and social media	148	40
Private companies	93	25.1
Pesticide’s label	152	41.1

* More than one answer was allowed; percentages of categories do not add up to 100.

Respondents’ purchasing pattern of fraudulent pesticides.

**Fig 1:**
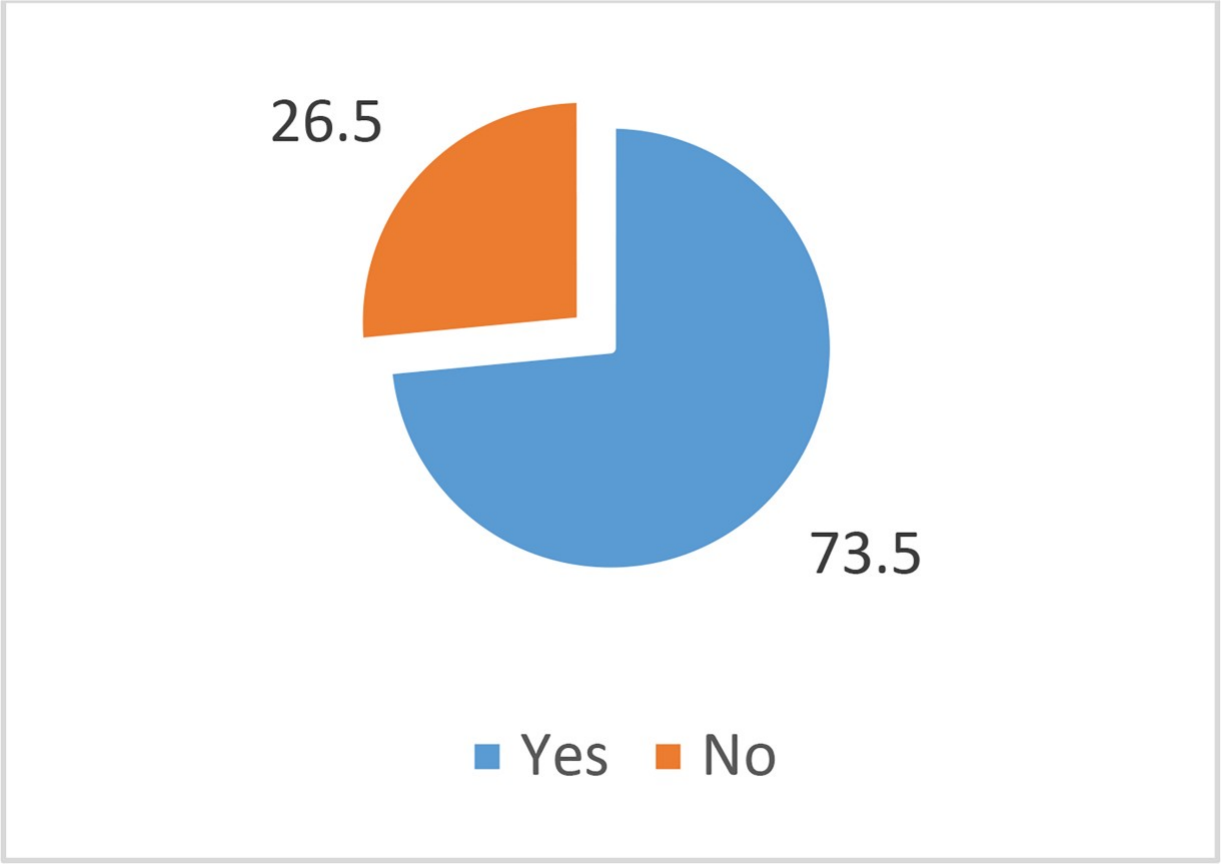
Purchasing fraudulent pesticides.

**Fig 2:**
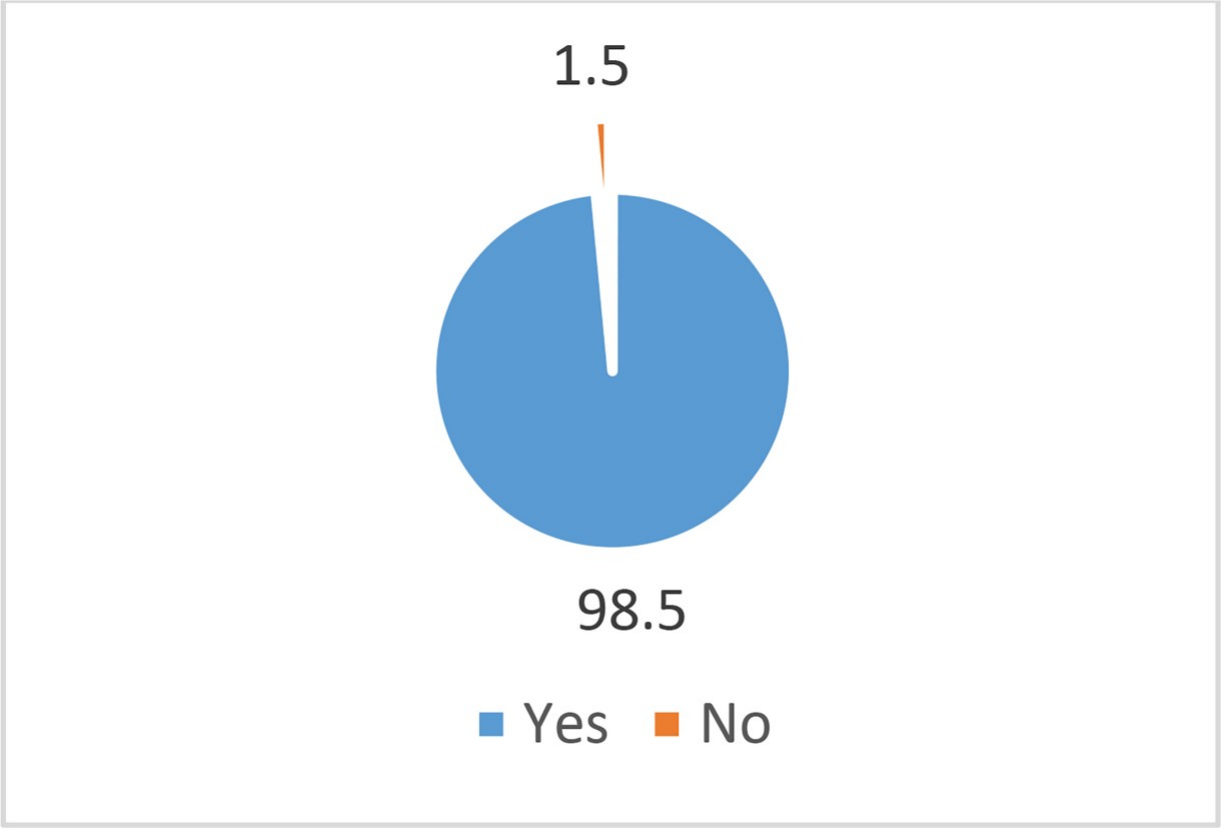
Purchasing fraudulent pesticides considering them genuine.

**Fig 3:**
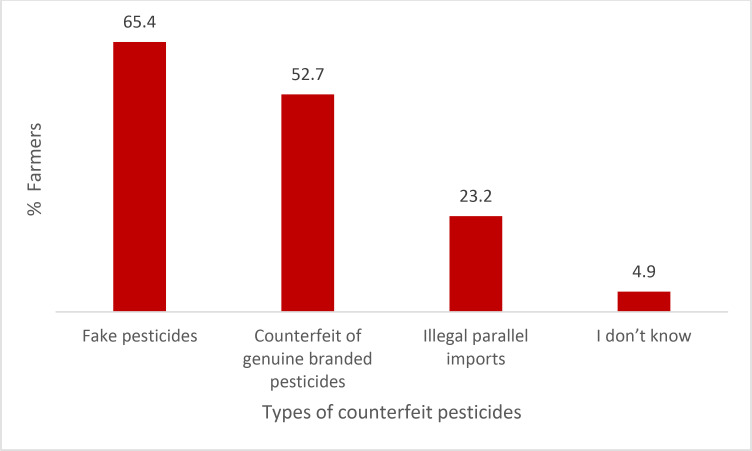
Types of fraudulent pesticides purchased (farmers who had purchased fraudulent pesticides).

A majority of the respondents (73.5%) had experienced purchasing fraudulent pesticides in the last three years, while 26.6% of the respondents had no experience in purchasing fraudulent pesticides (Fig 1). The results also showed that an overwhelming number of the respondents (98.5%) had purchased fraudulent pesticides as genuine products (Fig 2). In terms of the fraudulent pesticides purchased, as shown in Fig 3, the findings indicated farmers’ exposure to different types of fraudulent pesticides. Fake pesticides were the most popular choice among the other types. Almost two-thirds of the respondents (65.4%) had purchased pesticides sold in simple packs with minimal information on the label about their use and no mention of any environmental or health precautions. The next type mostly purchased by respondents was a sophisticated copy of a legitimate branded product, usually with a high quality of labeling and packaging. Such pesticides had been purchased by more than one-half of the respondents (52.7%). Besides that, legitimate parallel traded products, repackaged and sold as legitimate products, were also purchased by 23.2% of the respondents.

### Farmers’ perception of risks

Table 2 shows farmers’ perception of the risks associated with fraudulent pesticides. Overall, farmers had a moderate perception of the risks (mean = 3.88; standard deviation = 0.79). Details of each risk associated with fraudulent pesticides are provided below.

**Table 2:** Farmers’ perception of risks associated with fraudulent pesticides.

Variable	Statements	Mean	SD
Physical risk (M = 4.02, SD = 0.72)	I think that fraudulent pesticides can be dangerous for farmers’ health.	4.11	0.61
	I think that fraudulent pesticides can be dangerous for consumers’ health.	4.13	0.62
	I think that subsequent consequences of fraudulent pesticides hurt all wildlife and animals.	3.84	0.64
Agri-environmental risk (M = 3.47, SD = 0.72)	Loss or damage to the crop due to poor effect on pests.	3.43	0.87
	Loss or damage to the crop due to adverse physical effects of the product.	3.57	0.91
	Environmental contamination of soil and water from the unknown toxic effects of the product.	3.41	0.69
Economic risk (M = 3.52, SD = 1.11)	Loss of income from both crop loss or from damage, and from the cost of purchasing and applying the product.	4.04	0.69
	Buyers of export crops may refuse to deal with farmers, farmer’s marketing organisations, or produce from the country due to illegal residues.	3.59	1.23
	I think fraudulent pesticides reduce the sales of companies that market the original brands.	3.05	1.31
	I think that because of fraudulent pesticides, brands lose control of their reputation.	3.18	1.22
Social risk (M = 4.09, SD = 0.77)	I don’t like buying fraudulent pesticides because it gives other people a bad image of me.	4.17	0.58
	I don’t like buying fraudulent pesticides because I’m afraid that other people will notice it.	3.7	1.1
	I don’t like buying fraudulent pesticides because it is unethical.	4.04	0.84
	I would loss trust and respect of other people If I bought a fraudulent pesticide.	4.2	0.67
Legal risk (M = 4.31, SD = 0.68)	Closure of the business, fines, or a prison sentence due to breaking the law of the country.	4.36	0.64
	I don’t like buying fraudulent pesticides because I’m afraid of getting stopped at customs.	4.31	0.64
	Fraudulent pesticides infringe intellectual property rights.	4.25	0.78
Psychological risk (M = 4.1, SD = 0.63)	Buying fraudulent pesticides gives me a bad conscience.	4.17	0.57
	If I bought a fraudulent pesticide, I would feel guilty.	4.1	0.59
	If I bought a fraudulent pesticide, I would always blame myself.	4.02	0.73
Overall mean		3.88	0.79

### Physical risk

The perceived physical risk includes the threat to the health and safety of farmers and consumers. The mean values of the perceived physical risk ranged from 3.84 to 4.13, with an overall mean average of 4.02 (Table 2), indicating a high level of perception from the farmers’ perspective. The farmers rated fraudulent pesticides to be dangerous for consumers’ health as having the highest level of perception (mean = 3.46, SD = 0.71). This ranking of perception was followed by fraudulent pesticides can be dangerous for farmers’ health (mean = 4.11), and subsequent consequences of fraudulent pesticides hurt all wildlife and animals (mean = 3.84).

### Agri-environmental risk

The perceived agri-environmental risk includes the threat to crops, water, and soil. The findings in Table 2 show that the majority of respondents had a moderate perception of agri-environmental risks, with an overall mean average of 3.47. Table 2 highlights the areas causing a medium level of perception regarding fraudulent pesticides. These were loss or damage to the crop due to adverse physical effects of the product (mean = 3.43, SD = 0.92), loss or damage to the crop due to a poor effect on pests (mean = 3.57, SD = 0.87), and the environmental contamination of soil and water from unknown toxic effects of the product (mean = 3.41, SD = 0.69).

### Economic risk

Table 2 presents the means and standard deviations of farmers’ opinions on the perception of economic risks. The economic impact resulting in increasing trade of fraudulent pesticides is crucial for the industry of pesticides and the overall economic growth of society. As shown in Table 3, respondents had a moderate perception of the economic risks of fraudulent pesticides based on the overall mean score of 3.52. Considering all three statements assessed (Table 2), respondents considered the perception of economic risks as moderate.

**Table 3:** Differences between farmers who had purchased fraudulent pesticides and who had not, regarding their perception.

Variable	Mean	SD	t-value	p-value
Physical risk				
Not-purchased fraudulent pesticides	3.95	0.81	2.85**	0.005
Purchased fraudulent pesticides	3.81	0.94		
Agri-environmental risk				
Not-purchased fraudulent pesticides	3.77	0.93	-2.32*	0.02
Purchased fraudulent pesticides	3.92	0.83		
Economic risk				
Not-purchased fraudulent pesticides	3.25	0.68	-3.87**	0.00
Purchased fraudulent pesticides	3.62	0.74		
Social risk				
Not-purchased fraudulent pesticides	3.89	0.84	-2.24*	0.02
Purchased fraudulent pesticides	4.08	0.95		
Legal risk				
Not-purchased fraudulent pesticides	4.21	0.71	-2.07*	0.04
Purchased fraudulent pesticides	4.39	0.46		
Psychological risk				
Not-purchased fraudulent pesticides	3.86	0.66	-4.72**	0.00
Purchased fraudulent pesticides	4.65	0.53		

* P < 0.05

** P < 0.01.

### Social risk

The perceived social risk confronts farmers with negative reactions or thoughts of other people. The perceived psychological risk includes concerns about farmers' self-concept. The overall mean for the perception of social risk in our study was 4.09 (Table 2), indicating that farmers had a high perception of fraudulent pesticides in terms of their social risk. Obviously, the assessment of the two statements pertaining to the social risks (Table 2) shows that respondents considered the perception of social risks associated with fraudulent pesticides to be at a high level, while they had a lower perception of the other statement—“I don’t like buying fraudulent pesticides because I’m afraid that other people will notice it”—which they indicated as having a medium level of perception (mean = 3.7, SD = 1.1).

### Legal risk

The perception of legal risk is important, as it disables farmers from purchasing fraudulent pesticides in their farming context. The results shown in Table 2 indicate that farmers had a high perception of the legal risks (mean = 4.31). The statements with the highest legal risk ranked in order of perception (Table 3) were closure of the business, fines, or a prison sentence due to breaking the law of the country (mean = 4.36, SD = 0.64); “I don’t like buying fraudulent pesticides because I’m afraid of getting stopped at customs” (mean = 4.31, SD = 0.64); and fraudulent pesticides infringe intellectual property rights (mean = 4.25, SD = 0.78).

### Psychological risk

The perceived psychological risk includes concerns about farmers' self-concept. The overall mean for perceived psychological risks in our study was 4.1 (Table 2), indicating that farmers had a high perception of psychological risks associated with fraudulent pesticides. All three statements assessed were considered as being a high perceived psychological risk regarding fraudulent pesticides.

The results in Table 3 also revealed that there are significant differences between farmers who had purchased fraudulent pesticides and farmers who had not, at the level of at least 0.05, regarding all areas of risk under investigation. Farmers who had not purchased fraudulent pesticides had a higher perception regarding physical risk compared to non-purchased farmers (t = 2.85, P < 0.01). Conversely, farmers who had purchased fraudulent pesticides had a higher perception of the other risks associated with fraudulent pesticides.

### Factors influencing risk perception

As shown in Table 4, five of the six variables exhibit a significant relationship. Age (β = 0.25, p < 0.01), farm size (β = 0.29, p < 0.01), and extension as a source of information (β = 0.18, p < 0.01) have a positive relationship with farmers’ perception of the risks associated with counterfeit pesticides. Conversely, variables of farming experience (β = -0.34, p < 0.01) and purchased counterfeit pesticides (β = -0.11, p < 0.01) have a negative relationship with farmers’ perception. The findings also showed that the education level of farmers does not exhibit a significant relationship. The coefficient of multiple determination (R2) was 0.31. This denotes that approximately 31% of the total variation in farmers’ perception can be explained by the independent variables investigated.

**Table 4:** Results of the multivariate linear regression.

Variables	Unstandardized coefficients		Standardized coefficients	T	Collinearity statistics	
	B	S.E	Beta		TOL	VIF
Constant	71.38	2.44		29.22**		
Age	0.21	0.05	0.25	4.1**	0.52	1.91
Education	1.11	0.76	0.06	1.45	0.9	1.11
Farm size	0.02	0.003	0.29	6.21**	0.88	1.13
Farming experience	-0.26	0.05	-0.34	-5.27**	0.51	1.98
Extension as a source of information	3.07	0.79	0.18	3.84**	0.85	1.17
Purchased fraudulent pesticides	-2.03	0.84	-0.11	-2.42**	0.89	1.12

F = 27.18**, R = 0.55, R2 = 0.31, and

** P < 0.01.

## Discussion

The quality and authenticity of agricultural inputs are always a concern for farmers in different countries for increasing productivity and quality improvement. The issue of the presence of fraudulent pesticides and their impacts at micro and macro levels has received much attention in the literature. However, the perceptions regarding the adverse consequences of purchasing fraudulent pesticides can help explain adoption decisions. The present study is one of the first to study the perceptions of farmers regarding risks associated with fraudulent pesticides in the context of Saudi Arabia.

The results highlight the growing number of fraudulent pesticides in the study area. The majority of respondents expressed that they had purchased fake and illegal pesticides. Among them, the vast majority of respondents suffered from non-deceptive purchasing. The results are in agreement with the results of Haggblade et al. 2019A [34]. They conducted market survey of fraudulent pesticides in Mali and found that illegal and unregistered pesticides account for about 26% of all pesticide volumes sold. They confirmed the importance of the regional collaboration with neighbors across the region to control frauds. This result might be because the respondents relied upon other farmers and pesticide retailers for seeking information about pesticides (Table 1). The capability of farmers to give information to other farmers is determined by their recognizing behavior to detect fraudulent pesticides. This might be difficult to depend it on, in particular, under the continuous improvement of counterfeiting methods in recent years. Although pesticide retailers play a vital role in advising farmers about pesticide selection and handling during the purchase itself [35], some pesticide retailers may not be considered as a trusted source for information access. In reviewing the report issued by the ministry of commerce, one of the main drivers of fraudulent pesticides in Saudi Arabia is unauthorized shops [3]. Additionally, during the field study, we noticed that some retailers offer pesticides to farmers on a credit basis. This tendency may encourage farmers to trust pesticide retailers.

One of the positive trends observed in the current study is the good level of the farmers’ perception associated with fraudulent pesticides, where a majority confirmed the adverse consequences of these pesticides on different aspects of life. According to Pueschel et al. (2017) [21], risk perception generates cognitive dissonance and drives people to develop alternative strategies. In the context of frauds, high risk perception enables farmers to protect themselves by purchasing only products duly registered by governmental regulators [36]. This result is in line with the study of Ashour et al. (2019) [8], who revealed that 80% of the farmers in Uganda had high beliefs about counterfeit herbicides. On the other hand, a comparison of farmers who had purchased fraudulent pesticides and those who had not showed an interesting result. Farmers who purchased fraudulent pesticides had a higher perception regarding the majority of risk areas. A probable explanation for this result is that farmers who had purchased fraudulent pesticides noticed their adverse impact on the loss/damage of crops or ineffectiveness in pest control in their fields, as well as financial risk. Accordingly, they might be more aware of risks of fraudulent pesticides compared to farmers who have not purchased them before. This result is supported by the study of Ashour et al. (2019) [8], who reported that farmers who had purchased counterfeit herbicides had a high awareness of their risks and 31% of them had avoided purchasing herbicides in the next year from the same sources because of counterfeiting.

Seeking information from extension was found to be a significant predictor in the perception model. In other words, farmers who have relied upon extension to obtain information about fraudulent pesticides show a higher perception regarding the adverse effects of fraudulent pesticides than those who have not contacted with extension. Extension plays a crucial role in the dissemination of accurate information about pesticides, attitude reinforcement, and adoption enhancement [37,38]. This role can be achieved by awareness creation through different communication methods, as well as ensuring that stakeholders can recognize counterfeits and illegal pesticides by preparing extension publication and organizing training programs [34]. These results are consistent with the study of Jallow et al., (2017) [39], who reported that extension contact significantly influenced farmers’ risk perception of pesticide use in Kuwait. Similarly, in the current study, the respondents’ farm size significantly influenced the perception.

Specifically, increasing the size of the farm was associated with a high level of risk perception of fraudulent pesticides. A larger farm size farm usually implies greater overall economic losses if crops are damaged, forcing larger-scale farmers to purchase high-quality inputs [40]. This suggests that a larger farm size is conducive to farmers’ perception of fraudulent pesticides, whereas a smaller farm size may increase the risk of purchasing fraudulent pesticides in Saudi Arabia. Additionally, the results revealed that age is a significant determinant for risk perception. This means that older farmers in the study area showed higher levels of risk perception due to adverse effects of fraudulent pesticides. As indicated by Bonem et al. (2015) [41], older farmers perceived more risk and rated themselves as less likely to engage in risky behaviors than young farmers. Despite the importance of farming experience in reducing farmers’ exposure to economic risks and the efficient use of resources [42], its effect on farmers’ risk perception was negatively significant. A probable explanation for this result is that the risks were perceived as the highest by those farmers considered more vulnerable, such as less experienced farmers. Therefore, they pay more attention to acquiring accurate knowledge on pesticides to preserve their financial situation.

However, our study had some limitations that should be noted. First, we collected data from a random sample of farmers from one governorate in the eastern region of Saudi Arabia, so we could not generalize the results to other regions or other countries. Second, we were unable to assess whether farmers suffered from fraudulent pesticides. The farmers mentioned what they believed to be true, which could bias the results of the current situation of exposure to fraudulent pesticides. Third, we used a dichotomous question (yes/no) to describe purchasing behavior, which was not accurate enough to clarify the continuity of purchasing. This may lead to an inability to differentiate between a farmer who has purchased once and a farmer who has purchased many times. Therefore, exposure attributes of fraudulent pesticides should be clarified in further research, such as the frequency of exposure during a specific period, the timeliness of recognizing, and the behavior after recognizing to provide a full picture on farmers’ perception.

## Conclusion

In this study, we investigated the perception of risks associated with fraudulent pesticides among Saudi Arabian farmers. As this topic is limited in the literature, this study contributes to the existing body of knowledge by highlighting the relationship between purchasing fraudulent pesticides and perception, as well as factors influencing perception. We found that, despite the high percentage of fraudulent pesticides purchased, the respondents’ perception was above average for the risk areas of fraudulent pesticides. We also found that perception was positively associated with age, farm size, and extension and negatively associated with farming experience and the purchase of fraudulent pesticides. The findings provide useful directions and implications for policy makers. For example, the findings emphasize the growing number of fraudulent pesticides purchased by farmers in the study area. Hence, managers need to develop anti-counterfeiting procedures by enhancing coordination and integration among stakeholders across pesticides’ value chain. Furthermore, the findings of this research showed that that increasing perception is not sufficient for combating fraudulent pesticides. Therefore, complementary ICT-based extension campaigns (particularly those that allow both verbal and visual communication) have the great potential to raise awareness of recognizing fraudulent pesticides and fighting against such pesticides. Future research should examine the relationship between farmers’ exposure to fraudulent pesticides and their recognizing behavior. Furthermore, exploring drivers of farmers’ intention of use of such pesticides should also be considered.
